# Apolipoprotein-E forms dimers in human frontal cortex and hippocampus

**DOI:** 10.1186/1471-2202-11-23

**Published:** 2010-02-20

**Authors:** David A Elliott, Glenda M Halliday, Brett Garner

**Affiliations:** 1 Prince of Wales Medical Research Institute, Randwick NSW 2031, Australia; 2 School of Medical Sciences, Faculty of Medicine, University of New South Wales, Sydney NSW 2052, Australia; 3 School of Biological Sciences, University of Wollongong, Wollongong NSW 2522, Australia

## Abstract

**Background:**

Apolipoprotein-E (apoE) plays important roles in neurobiology and the apoE4 isoform increases risk for Alzheimer's disease (AD). ApoE3 and apoE2 are known to form disulphide-linked dimers in plasma and cerebrospinal fluid whereas apoE4 cannot form these dimers as it lacks a cysteine residue. Previous in vitro research indicates dimerisation of apoE3 has a significant impact on its functions related to cholesterol homeostasis and amyloid-beta peptide degradation. The possible occurrence of apoE dimers in cortical tissues has not been examined and was therefore assessed. Human frontal cortex and hippocampus from control and AD post-mortem samples were homogenised and analysed for apoE by western blotting under both reducing and non-reducing conditions.

**Results:**

In apoE3 homozygous samples, ~12% of apoE was present as a homodimer and ~2% was detected as a 43 kDa heterodimer. The level of dimerisation was not significantly different when control and AD samples were compared. As expected, these dimerised forms of apoE were not detected in apoE4 homozygous samples but were detected in apoE3/4 heterozygotes at a level approximately 60% lower than seen in the apoE3 homozygous samples. Similar apoE3 dimers were also detected in lysates of SK-N-SH neuroblastoma cells and in freshly prepared rabbit brain homogenates. The addition of the thiol trapping agent, iodoacetamide, to block reactive thiols during both human and rabbit brain sample homogenisation and processing did not reduce the amount of apoE homodimer recovered. These data indicate that the apoE dimers we detected in the human brain are not likely to be post-mortem artefacts.

**Conclusion:**

The identification of disulphide-linked apoE dimers in human cortical and hippocampal tissues represents a distinct structural difference between the apoE3 and apoE4 isoforms that may have functional consequences.

## Background

Apolipoprotein-E (apoE) is a ~34 kDa protein that plays important roles in lipid transport and neurobiology [1,2]. In humans, apoE exists as three major isoforms apoE2, apoE3 and apoE4 which differ in their Cys/Arg composition at positions 112 and 158. ApoE2 contains Cys^112^, Cys^158^; apoE3 contains Cys^112^, Arg^158^; and apoE4 contains Arg^112^, Arg^158 ^[3]. ApoE4 is a major genetic risk factor for late-onset Alzheimer's disease (AD) whereas apoE2 is associated with decreased AD risk [4,5]. ApoE in the CNS is primarily produced by astrocytes, although microglia and neurons may also contribute under certain circumstances [1,6-11].

ApoE participates in several biological processes that extend beyond lipid transport and include immunoregulation, oxidative stress, stabilization of neuronal microtubules, nerve regeneration, apoptosis and amyloid-beta (Aβ) peptide clearance and degradation [11-20]. Despite intense research into the diverse biological functions of apoE, the precise mechanism by which the apoE4 isoform increases AD risk remains to be fully elucidated. However, many differences between apoE3 and apoE4 structure and function have been reported that are potentially relevant to AD. These include: reduced lipid-binding capacity of apoE4 due to isoform-specific domain interactions [21], lipidated apoE4 has a lower affinity for Aβ [22,23], apoE4 is less efficient at stabilizing microtubules [14], apoE4 exhibits weaker antioxidant activity [13] and apoE4 is structurally less stable [24,25] when compared to apoE3. It is also clear that the proteolytic fragmentation of apoE in the human brain is isoform-dependent [26-29]. In addition, a significant proportion of apoE3 (and apoE2) is present in plasma, CSF and astrocyte conditioned media as a disulfide-linked homodimer and as an apoE-apoA-II heterodimer [30-34]. This may be important as apoE4 lacks Cys and cannot form disulphide-linked dimers. In vitro studies have shown that compared to apoE monomers, apoE dimers possess significantly altered functional properties in terms of their capacity to regulate cellular cholesterol efflux and to interact with Aβ [30,35-38]. This further underscores the importance of probing for the possible occurrence of apoE dimers in the human brain. In the present study we demonstrate that disulphide-linked apoE homodimers and heterodimers are present in the human cortex and hippocampus. In addition, we show that apoE dimerisation was not affected by the presence of AD.

## Results

### ApoE3 forms disulfide-linked dimers in the human brain

**Figure 1:**
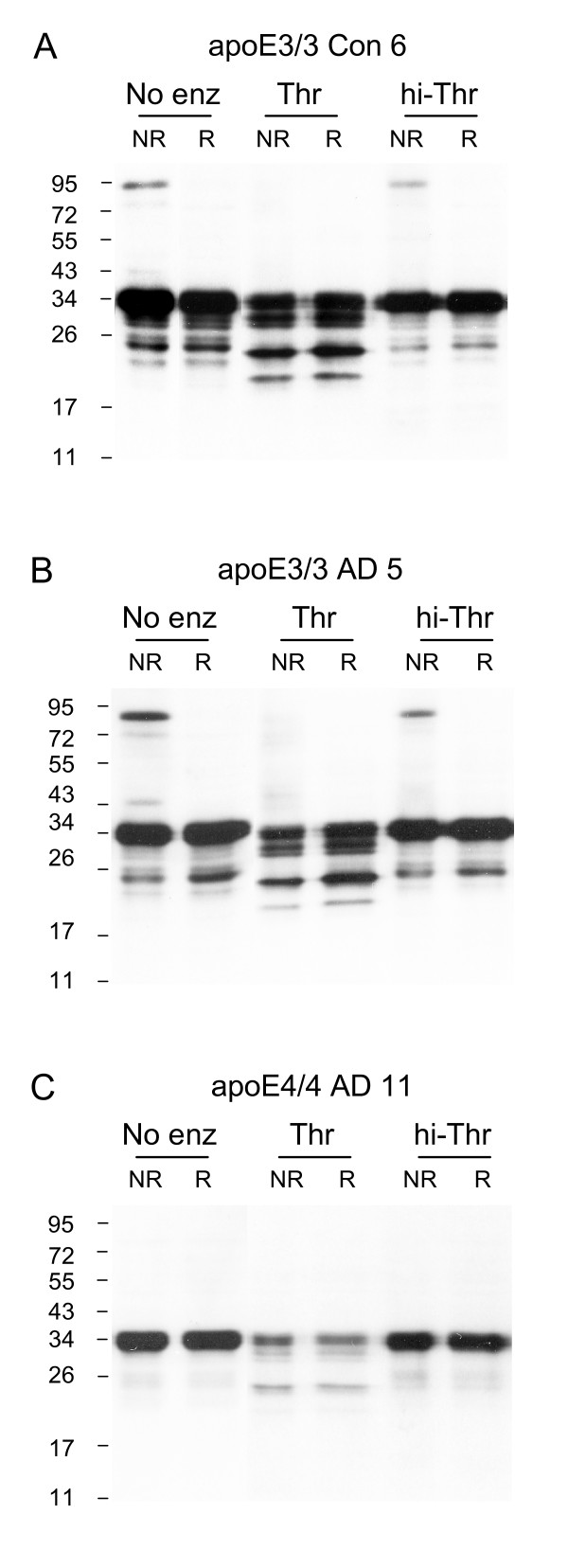
**ApoE3 dimers are present in human hippocampus**. The presence of disulphide-linked dimers of apoE were detected by analysing samples under both non-reducing "NR" and reducing "R" SDS-PAGE conditions. The susceptibility of dimers to thrombin cleavage was tested using three different conditions; storage at -80°C with no enzyme added "No enz", incubation at 37°C in the presence of thrombin "Thr" or heat-inactivated thrombin "hi-Thr". TBS-soluble fractions from control apoE3 (A), AD apoE3 (B) and AD apoE4 (C) homozygous hippocampal tissue samples were analysed. Western blotting was performed using goat anti-apoE polyclonal antibody. The human brain samples (Con n = 1, AD n = 2) are identified according to the Case # code given in Table 1.

It is established that apoE3 forms disulphide-linked homodimers and apoE3-apoA-II heterodimers in human plasma and CSF [30,33]. As apoE4 lacks Cys it cannot form disulphide bonds. Whether apoE3 exists in a dimeric state in human brain tissue is unknown and we therefore focused on this issue. Western blot analysis of TBS-soluble brain homogenates derived from the hippocampus of a control apoE3/3 subject indicated a clear apoE3 homodimer when the sample was run under non-reducing conditions (Fig 1A). The homodimer was detected at ~95 kDa (as opposed to the predicted ~68 kDa) which is consistent with previous data [30,33]. ApoE contains a major cut site for thrombin in the linker region [39]. Due to the high proteolytic specificity of thrombin [40,41], it was used here to further confirm the identification of the ~95 kDa band as an apoE homodimer. The putative apoE3 homodimer was removed after incubation with thrombin, as predicted, further confirming that the ~95 kDa band is not likely to be due to non-specific binding of the antibody used.

Incubation of the homogenate in the presence of heat-inactivated thrombin resulted in a partial loss of the apoE dimer which suggests that endogenous proteases may also degrade apoE (Fig 1A). A band of relatively lower intensity was also observed at ~43 kDa in the non-incubated apoE3 control condition. This is consistent with the apoE-apoA-II heterodimer previously detected in human plasma and CSF [30,33]. A series of apoE fragments was also detected with a major band at ~24 kDa. This is in close agreement to our previous observations [29].

**Figure 2:**
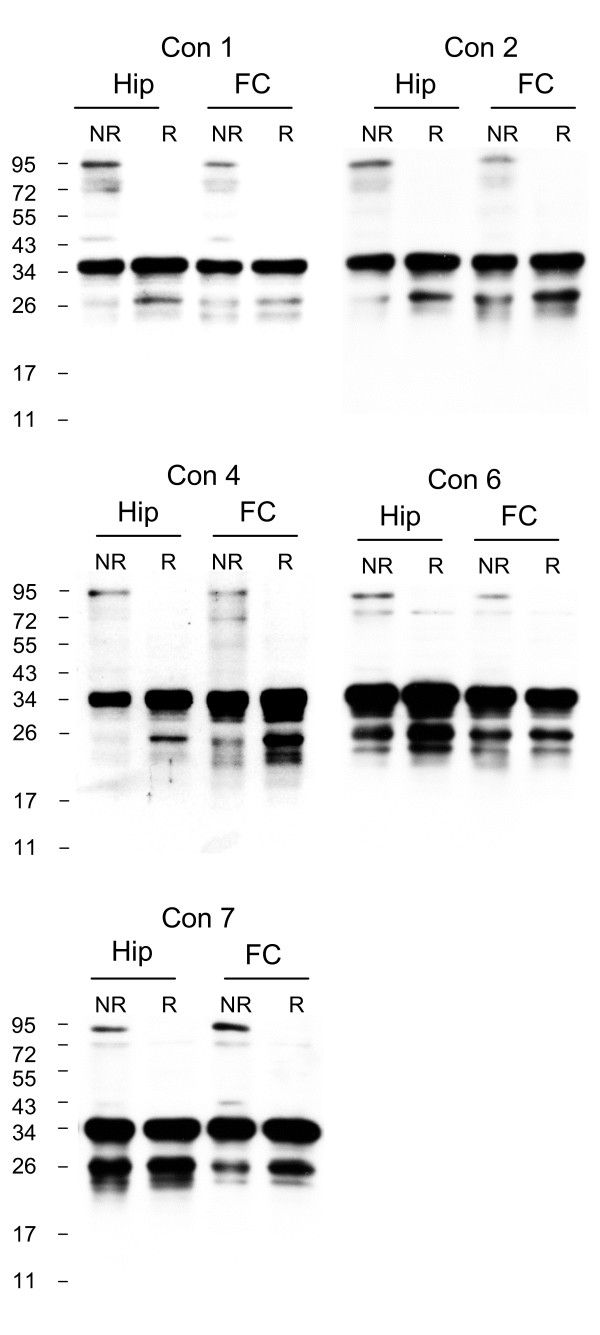
**ApoE3 dimers are present in both hippocampus and frontal cortex of control apoE 3/3 brain homogenates**. Comparisons were made between the hippocampus and frontal cortex in each individual control apoE3/3 brain, under non-reduced (NR) and reduced (R) conditions. Western blotting was performed using goat anti-apoE polyclonal antibody. The human brain samples (Con n = 5) are identified according to the Case # code given in Table 1.

Additional hippocampal and frontal cortex homogenates from control apoE3/3 donors were analysed and this revealed that the apoE3 homodimer was present in all samples and accounted for 8.3 ± 0.9% (mean ± SE, n = 6) of the total apoE present in the hippocampus and 16.5% ± 4.1% (mean ± SE, n = 7) of the total apoE present in the frontal cortex (Fig 2). Although the percentage of apoE present as the homodimer was on average increased in the frontal cortex, this difference did not reach statistical significance. We also analysed frontal cortex and hippocampus derived from apoE3/3 AD samples and found that apoE3 dimers were detected in all AD samples and were identical to those in the control samples (Fig 1B). There was no significant difference regarding the proportion of apoE that was present in the dimerised form when frontal cortex from the two groups were compared (Control 16.5 ± 4.1%, mean ± SE, n = 7; AD 11.2 ± 2.4%, mean ± SE, n = 5).

**Figure 3:**
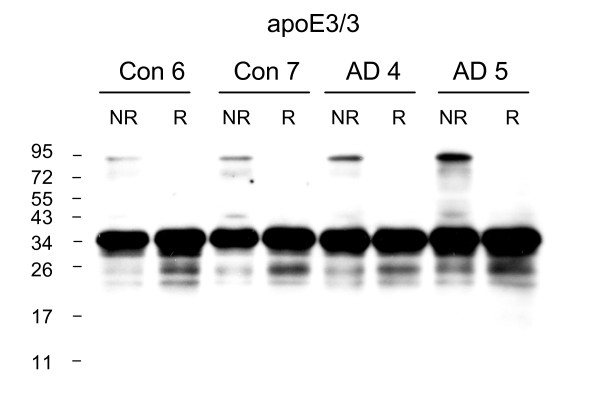
**ApoE3 dimers are present in the detergent (Triton-X-100) soluble fraction of both control and AD brain homogenates**. The presence of apoE3 dimers was assessed in the Triton-X-100 soluble fraction of frontal cortex samples from control apoE3/3 and AD apoE3/3 brains, under non-reduced (NR) and reduced (R) conditions. Western blotting was performed using goat anti-apoE polyclonal antibody. The human brain samples (Con n = 2, AD n = 2) are identified according to the Case # code given in Table 1.

We also used an additional rigorous extraction protocol employing extraction buffer that contained the detergent Triton-X100. This was done in order to maximise recovery of apoE that may be associated with TBS-insoluble material. Both the control and AD samples were found to contain apoE homodimers when samples were extracted in detergent-containing buffer (Fig 3). These data are very similar to the results obtained with the TBS-soluble homogenates (Fig 1).

**Figure 4:**
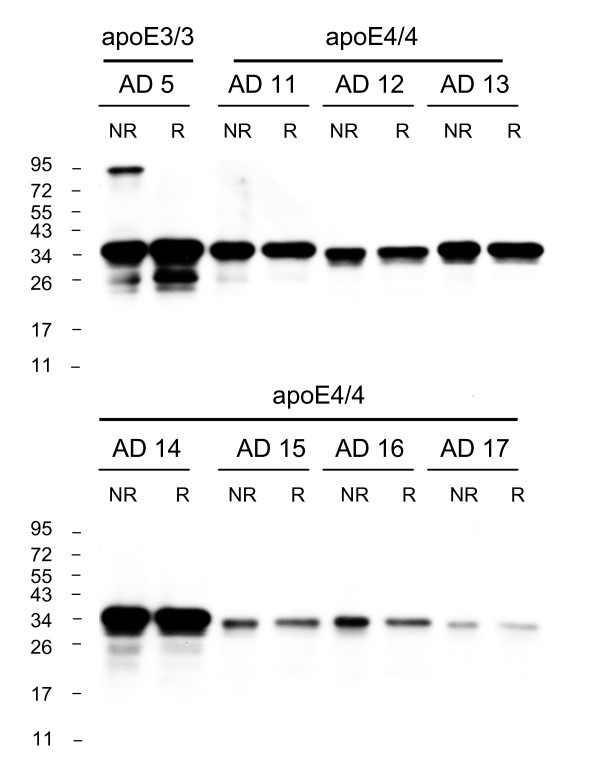
**ApoE dimers are absent in the AD apoE4/4 brain homogenates**. The absence of apoE dimers in AD apoe4/4 brains was confirmed in all available samples (n = 7) and compared to a reference AD apoE3/3 brain. Samples were analysed under non-reduced (NR) and reduced (R) conditions and western blotting was performed using goat anti-apoE polyclonal antibody. The human brain samples (AD n = 8) are identified according to the Case # code given in Table 1.

**Figure 5:**
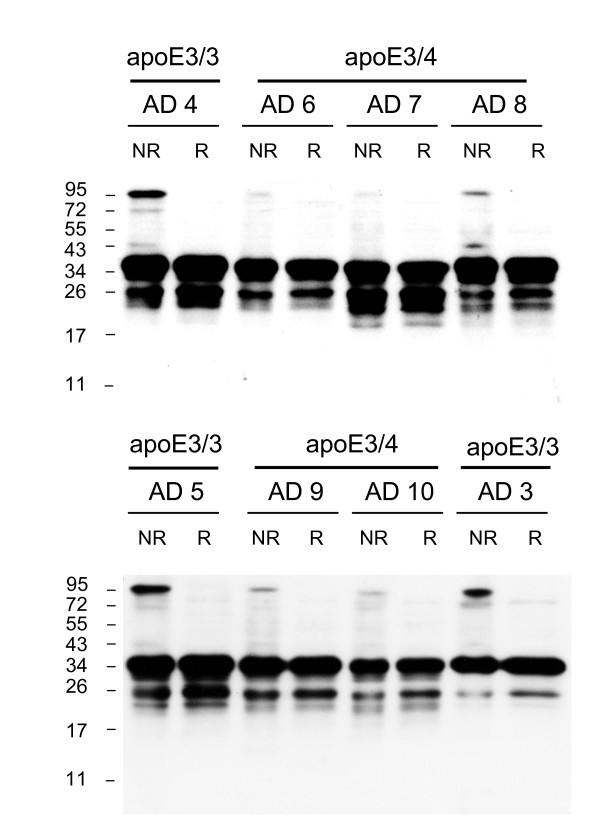
**Proportion of apoE present as dimers is significantly lower in heterozygous apoE3/4 AD brain homogenates**. The proportion of total apoE present as dimers was compared between homozygous apoE3/3 and heterozygous apoE3/4 frontal cortex homogenates, under non-reduced (NR) and reduced (R) conditions. Western blotting was performed using goat anti-apoE polyclonal antibody. The human brain samples (AD n = 8) are identified according to the Case # code given in Table 1.

As predicted, apoE dimers were not detected in any of the 7 apoE4/4 AD samples analyzed (Fig 4, see also Fig 1C). Analysis of heterozygous apoE3/4 AD samples revealed a significant 61% reduction in the percentage of apoE3 present as homodimer as compared to apoE3/3 homozygous AD samples (AD 3/3 11.2 ± 2.4%, mean ± SE, n = 5; AD 3/4 4.4% ± 0.6, mean ± SE, n = 5; p < 0.03). Representative blots from heterozygous apoE3/4 AD samples are shown (Fig 5).

### ApoE3 forms disulphide-linked dimers in human SK-N-SH neurons and rabbit brain

One potential issue that arises in the analysis of protein modifications in human post-mortem material is the potential for artifactual changes to be induced by post-mortem interval (PMI). Even though clear ~95 kDa and ~43 kDa apoE dimers were detected in apoE3 AD homozygous samples with a post-mortem delay of as short as 1 h (and PMI for the AD sample shown in Fig 1B was only 7 h), we also probed for apoE dimers in human neuroblastoma cell lysates and freshly harvested rabbit brain. The human SK-N-SH neuroblastoma cell line expresses the *APOE *ε3/ε3 genotype [42,43] and synthesizes large amounts of apoE under serum starved conditions [11], whereas rabbits are one of the few non-human species known to contain an apoE Cys^112 ^residue [31].

**Figure 6:**
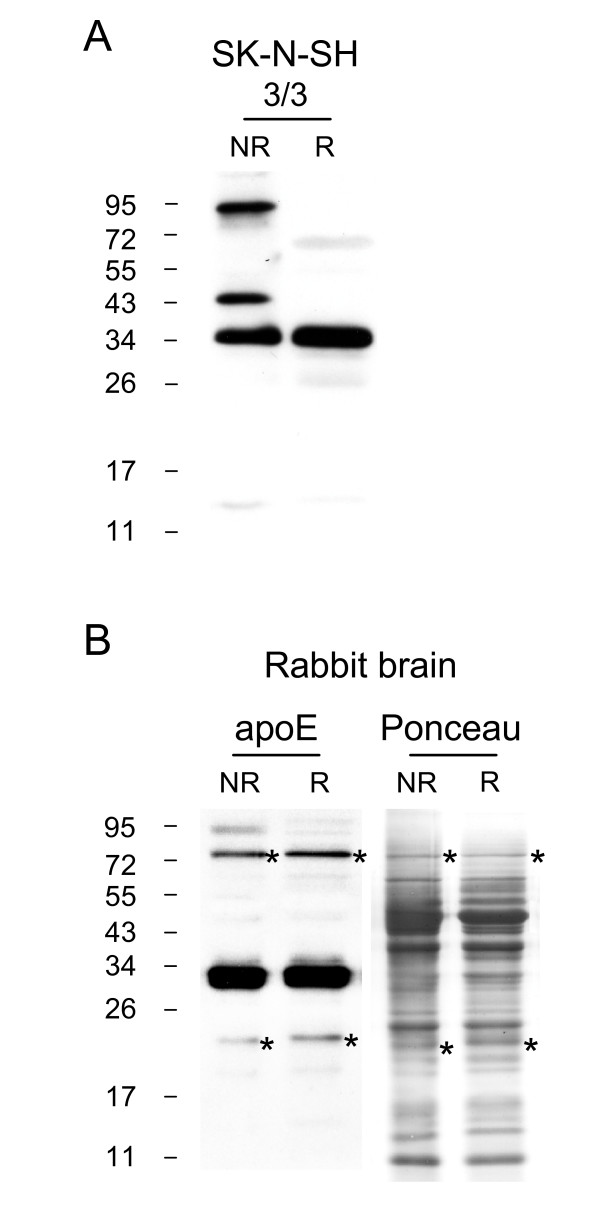
**ApoE3 dimers are present in SK-N-SH cell lysate and rabbit frontal cortex**. SK-N-SH cell lysate was analysed under non-reduced (NR) and reduced (R) SDS-PAGE conditions and apoE was detected using anti-apoE C-terminal monoclonal antibody (A). TBS-soluble rabbit brain homogenate was analysed under NR and R conditions and apoE detected using goat anti-apoE polyclonal antibody (B). Two bands marked with asterisks are believed to be due to non-specific cross-reaction with the proteins indicated (asterisks) by Ponceau staining (B).

Analysis of SK-N-SH cell lysates under non-reducing conditions revealed the presence of the ~95 kDa apoE homodimer and a more prominent (than human brain) ~43 kDa heterodimer (Fig 6A). Since the ~43 kDa dimer could theoretically represent a disulphide-linked apoE N-terminal domain homodimer (a predicted MW of ~44 kDa), we used a C-terminal specific monoclonal antibody in this experiment and the detection of the ~43 kDa band indicates an intact C-terminus. This rules out the presence of disulphide-linked N-terminal domain homodimer. The ~43 kDa band may represent an apoE3-apoA-II heterodimer as has been previously described in human plasma and CSF [30,33].

In the analysis of rabbit frontal cortex, the brain was dissected and processed immediately at the time of death to eliminate post-mortem delay and all measures were taken to prevent serum and CSF contamination (see Materials and Methods). An apoE band at ~95 kDa was also detected in rabbit brain when samples were run in the non-reduced state (Fig 6B), again indicating that apoE containing Cys^112 ^does form a homodimer in the brain.

**Figure 7:**
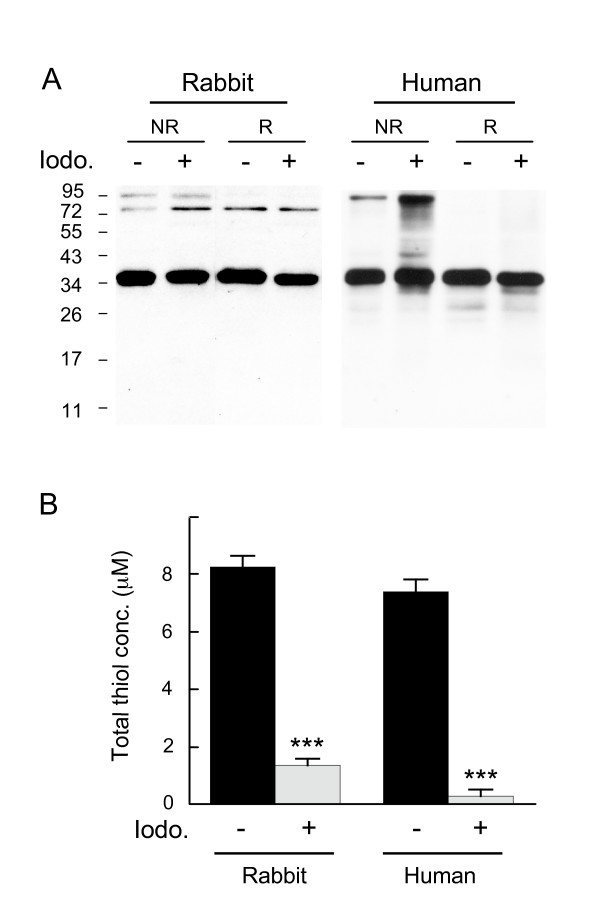
**ApoE dimer is not artificially formed during sample homogenisation and processing**. Freshly prepared rabbit and frozen human brain tissue (AD apoE3/3, AD 5) was homogenised in a detergent-rich lysis buffer either with or without the thiol-trapping agent iodoacetamide (Iodo) used at a final concentration of 100 mM. The effect of Iodo on the presence of apoE dimers was assessed under non-reduced (NR) and reduced (R) conditions by western blot, using goat anti-apoE polyclonal antibody (A). The extent of thiol-trapping by Iodo was determined by measuring the total thiol present, using DTNB assay (B). Data in B are means of triplicate readings with S.E. represented by error bars. *** *P *< 0.0001. The human brain sample (AD n = 1) is identified according to the Case # code given in Table 1.

To address the possibility that the observed apoE dimerisation may occur during tissue homogenization and processing for electrophoresis, freshly prepared rabbit brain and frozen human frontal cortex tissue (AD apoE3/3) were homogenized in buffer containing the thiol trapping agent iodoacetamide. Homogenization was also performed using a detergent-rich lysis buffer to delipidate apoE-containing lipoproteins and help prevent the possibility of dimers forming spontaneously on lipoprotein particles during homogenization and processing. The presence of 100 mM iodoacetamide did not result in a decrease in apoE homodimer levels in either rabbit or human brain tissue (Fig 7A). The thiol-trapping efficiency of 100 mM iodoacetamide was confirmed by spectrophotometric analysis of the total thiol concentration of the homogenates using Ellman's reagent (5,5'-Dithio-bis(2-nitrobenzoic acid)). The total thiol concentration was significantly reduced in both rabbit and human brain homogenates (84% and 96%, respectively) treated with iodoacetamide (Fig 7B). This data demonstrates that apoE dimers are not artificially formed during tissue homogenization and processing, and further indicates that apoE dimers are generated in the brain.

## Discussion

This study demonstrates for the first time that apoE3 disulphide-linked dimers are present in human frontal cortex and hippocampus. Furthermore, strikingly similar dimers were also detected in human neuroblastoma cells expressing apoE3 and in freshly prepared rabbit brain. These data indicate that apoE3 dimerisation is a physiologically relevant process in the human brain; as it is in human CSF and plasma [30,33]. The biological functions of these apoE dimers in the brain is not yet clear, however, several studies support the possibility that they may have distinct properties that differ from apoE monomer. Previous studies have demonstrated that apoE3 homodimers and apoE3-apoA-II heterodimers have diminished low-density lipoprotein receptor binding activity (20% and 30%, respectively) in comparison to apoE3 monomer [30,35]. In vitro studies have shown that the apoE-apoA-II dimer is more effective than apoE monomer in binding soluble Aβ1-42 and inhibiting its internalisation by neurons [36,37]. In addition, a recent study has demonstrated that the apoE3 homodimer is more effective than monomeric apoE3 at enhancing ABCA1-dependent lipid efflux from neurons [38].

Based on these data, it seems possible that the reported physiological properties of apoE dimers may enhance some of the AD-protective functions attributed to apoE3. For example, the role of apoE in Aβ clearance and degradation [18,20] may be facilitated by enhanced binding with Aβ1-42 [37]. In addition, the enhanced neuronal cholesterol efflux that is induced by apoE dimers as compared to monomers [38] may be important considering that high levels of neuronal cholesterol can influence processing of the amyloid precursor protein and increase Aβ production [44-47]. Thus dimerisation may represent one mechanism by which apoE3 and apoE2 have a distinct AD-protective advantage over apoE4. However, this feature alone is probably not the crucial regulator underlying apoE genotype-associated risk as the dimers were detectable at similar levels in apoE3 homozygous control and AD samples.

## Conclusion

In conclusion, the presence of apoE3 dimers in the human brain represents a fundamental structural difference between apoE3 and apoE4. A greater understanding of the biological consequences of this difference may shed light on the isoform-dependent influences of apoE on AD risk.

## Methods

### Human brain tissue

Brain tissue samples were obtained through the Australian Brain Donor Program with ethics approval from the University of New South Wales Human Research Ethics Committee (approval No. HREC03322). The research was carried out in compliance with the Helsinki Declaration. Cortical neuritic plaques and neurofibrillary tangles were assessed according to current international standards in order to pathologically confirm the diagnosis of AD post-mortem [48,49]. Sample details are provided in Table 1.

**Table 1:** Brain donor information

Case #	Diagnosis	Sex	APOE genotype	Age at death	PMI	Brain regions analysed	ApoE homodimer as % of total apoE
CON 1	Normal	F	ϵ3/ϵ3	73	60	FC; H	23.5; 11.4
CON 2	Normal	F	ϵ3/ϵ3	83	24	FC; H	21.3; 9.1
CON 3	Normal	F	ϵ3/ϵ3	77	36	FC	4.2
CON 4	Normal	M	ϵ3/ϵ3	79	60	FC; H	16.7; 10
CON 5	Normal	M	ϵ3/ϵ3	82	43	FC; H	33.4; 6.6
CON 6	Normal	F	ϵ3/ϵ3	93	21	FC; H	2.47; 6.15
CON 7	Normal	F	ϵ3/ϵ3	85	23	FC; H	13.73; 6.75
AD 1	AD	F	ϵ3/ϵ3	79	4	FC	4.4
AD 2	AD	M	ϵ3/ϵ3	60	2	FC	10.4
AD 3	AD	M	ϵ3/ϵ3	75	1	FC	19.13
AD 4	AD	M	ϵ3/ϵ3	70	35	FC; H	12.55; 7.56
AD 5	AD	F	ϵ3/ϵ3	94	7	FC; H	9.54; 12.16
AD 6	AD	F	ϵ3/ϵ4	83	3	FC	3.61
AD 7	AD	M	ϵ3/ϵ4	73	16	FC	2.28
AD 8	FAD	F	ϵ3/ϵ4	47	69	FC	4.94
AD 9	FAD	M	ϵ3/ϵ4	51	5	FC	5.2
AD 10	AD	M	ϵ3/ϵ4	83	36	FC	5.88
AD 11	AD	M	ϵ4/ϵ4	74	5	FC; H	0
AD 12	AD	F	ϵ4/ϵ4	75	80	FC	0
AD 13	AD	F	ϵ4/ϵ4	68	44	FC	0
AD 14	AD	M	ϵ4/ϵ4	83	25	FC	0
AD 15	AD	F	ϵ4/ϵ4	78	24	FC	0
AD 16	AD	M	ϵ4/ϵ4	67	60	FC	0
AD 17	AD	F	ϵ4/ϵ4	84	74	FC	0

Brain donor information (AD, Alzheimer's disease; FAD, familial Alzheimer's disease; C, control; PMI, post-mortem interval (hours); H, hippocampus; FC, frontal cortex)

### Human tissue preparation

Samples were taken from the frontal cortex or hippocampus; both areas that are affected by AD [50]. The homogenisation protocol was previously described in detail [29], and was used for all samples unless stated otherwise. In brief, between 60-90 mg of brain tissue was homogenized with a pre-chilled 1 mL glass dounce homogenizer, using 15 volumes of ice-cold tris-buffered saline (TBS) pH 7.4. Protease and phosphatase inhibitors (Calbiochem, San Diego, USA) were added to all samples except those to be used in experiments requiring enzyme addition (samples C6, AD5 and AD6). After centrifugation at 16,000 g for 25 minutes at 4°C the TBS-soluble supernatant fraction was collected. Where specified, the pellet was then washed with 50 μL of TBS and centrifuged again for 5 min, the supernatant was then discarded and pellet was resuspended in 15 volumes of TBS containing protease and phosphatase inhibitors and 1% (w/v) Triton X-100 (TBS-X) by pipetting up and down. Samples were then mixed by rotation for 30 min at 4°C, followed by centrifugation at 16,000 g for 25 min at 4°C and collection of the TBS-X soluble supernatant fraction.

A second protocol was also used, where specified, to homogenise brain tissue in a detergent-rich lysis buffer. In brief, brain tissue was homogenized with a pre-chilled 1 mL glass dounce homogenizer, using 10 volumes of DRLB (50 mM Tris-HCl, pH 8.0, 150 mM NaCl, 0.1% SDS, 0.5% IGEPAL CA-630, 0.5% sodium deoxycholate) with protease and phosphatase inhibitors and either with or without 100 mM iodoacetamide (Sigma) to trap thiol groups and thus prevent changes in disulphide bond status during homogenization and sample processing [51]. Homogenates were then centrifuged at 16,000 g for 10 min at 4°C in an Eppendorf 5417-R refrigerated Microfuge and the supernatants collected.

### Rabbit tissue preparation

An adult male Watanabe rabbit was euthanased via cardiac puncture using 5 mL Lethabarb (1 mL per 2 kg body weight, Virbac, Sydney, Australia) and the brain surgically removed to dry ice and processed immediately in order to eliminate post-mortem delay. Approximately 100 mg of tissue was removed from the cerebral cortex, all visible vasculature was removed and the sample was rinsed three times in ice-cold phosphate-buffered saline (PBS). The sample was then homogenized following the protocol used above for the human samples.

### ApoE genotyping

Genomic DNA was extracted from brain tissue and *APOE *amplified by PCR. Briefly, each reaction (50 μL) contained 200 nM of each primer (Invitrogen, Carlsbad, CA) 5'-TCCAAGGAGCTGCAGGCGGCGCA-3' (forward) and 5'-ACAGAATTCGCCCCGGCCTGGTACACTGCCA-3' (reverse), 2 mM dNTPs, 2 mM MgCl2, 2 U Taq polymerase (PCR reagents supplied by Promega, Madison, WI) and 400 ng DNA, all combined in nuclease free H_2_O. Amplification was carried out with 38 cycles of denaturation (95°C, 30 sec), annealing (60°C, 30 sec) and extension (70°C, 30 sec). The 244 bp PCR product was purified using the QIAquick PCR purification kit (Qiagen, Venlo, Netherlands), following the manufacturer's protocol, and eluted in 40 μL H_2_O. Endonuclease restriction digests (25 μL) were performed on 15 μL of eluted DNA using either AflIII (5 U) or HaeII (20 U) in the presence of BSA (100 μg/mL) and the supplied buffer (New England Biolabs, Ipswich, MA) at 37°C for 16 h. The ε3 allele is resistant to both enzymes while ε4 is cleaved by AflIII (producing a 190 bp product) and ε2 is cleaved by HaeII (producing a 191 bp product) assessed using ethidium bromide stained 8% polyacrylamide gels.

### SK-N-SH neuroblastoma cell culture

Cell culture media and additives were from Invitrogen (Melbourne, Australia). Human neuronal SK-N-SH cells were routinely grown in DMEM, 10% (v/v) fetal calf serum (FCS), 2 mM glutamine, and 100 IU/ml penicillin and 100 μg/ml streptomycin. Cultures were grown in 75 cm^2 ^flasks at 37°C in 5% CO_2 _and plated into 6-well plates for use in experiments. To induce apoE expression, SK-N-SH cells were cultured under serum starved conditions (5 days of culture without media replenishment) and harvested in cell lysis buffer (10 mM Tris-HCl, 10 mM Na_2_PO_4_/NaHPO_4_, pH 7.5, 130 mM NaCl, 1% Triton-X-100, 10 mM NaPP_i_) as described previously [11]. SK-N-SH cells have been shown to be apoE3/3 homozygous [42,43].

### Western blotting

Bicinchoninic acid protein assays were performed on brain homogenate samples and equal amounts of protein were separated on 12% SDS-PAGE gels and transferred onto 0.45 μm nitrocellulose membranes at 100 V for 30 min. Membranes were Ponceau-stained and scanned before blocking overnight at 4°C in PBS containing 5% (w/v) non-fat dry milk. The membranes were then probed with the relevant antibodies at 22°C for 1 h to reveal the bands of interest. Concentrations of antibodies were: goat polyclonal anti-human apoE 1/5000 (Calbiochem) or mouse monoclonal anti-human apoE 21-F3-D2 1/1000 (Biogenesis, Poole, UK). The membranes were washed three times in PBS containing 0.1% (w/v) Tween-20 and then incubated with horseradish peroxidase-conjugated rabbit anti-goat (Dako, 1/2500) or rabbit anti-mouse (Dako, 1/1000) secondary antibody for 1 h. The proteins of interest were detected using enhanced chemiluminescence (ECL, Amersham Biosciences) and X-ray film. Signal intensity was quantified using Image-J software. Specifically, a fixed area was used to separately measure signal intensity from i) the region encompassing intact ~34 kDa and fragmented ~24 kDa apoE, ii) apoE homodimer at ~95 kDa, and iii) an adjacent blank region to serve as a background control. The background value (iii) was subtracted from the apoE measurements (i and ii) and the homodimer quantification was expressed as a percentage of total apoE. Where possible, relative differences between samples were assessed on the same blots or using simultaneously processed gels with identical film exposure times.

### Enzymatic treatment of brain homogenates

Thrombin digestion was performed by incubating brain homogenates (30 μg of protein) prepared in the absence of protease inhibitors with 4.5 U of thrombin (Sigma, St. Louis, MO) in PBS at 37°C for 16 hours. Two control conditions were also analysed: homogenates either stored at -80°C for the incubation period or incubated with thrombin that was heat-inactivated at 95°C for 15 minutes.

### Thiol quantification

The concentration of total thiol groups, both protein-bound and free, in specified brain homogenates was determined using 5,5'-dithio-bis(2-nitrobenzoic acid) (DTNB, Sigma) also known as 'Ellman's Reagent' as described previously [52]. Briefly, a 30 μL volume of brain homogenate (either undiluted or diluted in homogenisation buffer) was combined with 75 μL of dilution buffer (30 mM Tris HCl, 3 mM EDTA pH 8.2), 25 μL of DTNB reagent (1.19 mg/mL DTNB in methanol) and 400 μL methanol. Samples were centrifuged at 3000 g for 5 min at room temperature. 3 × 90 μL aliquots of supernatant were collected and absorbance was measured at 415 nm. Thiol concentration was determined by reference to a cysteine (Sigma) standard curve.

## Abbreviations

Aβ: amyloid-β; AD: Alzheimer's disease; apoE: apolipoprotein-E; CNS: central nervous system; Con: control; CSF: cerebrospinal fluid; DTNB: 5,5'-Dithio-bis(2-nitrobenzoic acid); HI-Thr: heat-inactivated thrombin; Iodo: iodoacetamide; NR: non-reduced; PAGE: polyacrylamide gel electrophoresis; PBS: phosphate buffered saline; PMI: post-mortem interval; R: reduced; SDS: sodium dodecyl sulfate; TBS: tris buffered saline; TBS-X: TBS containing 1% (w/v) Triton X-100; Thr: thrombin.

## Authors' contributions

DE carried out the experimental work, performed the statistical analysis and drafted the manuscript. GH collected and provided human brain tissues. BG conceived of the study, participated in its design and coordination and prepared the final manuscript. All authors read and approved the final manuscript.
